# Restriction times on the rise: mechanistic modelling of activity time of grassland vipers (*Vipera *spp*.*) in the face of climate change

**DOI:** 10.1186/s12983-025-00564-4

**Published:** 2025-06-23

**Authors:** Edvárd Mizsei, Tibor Sos, Attila Móré, Bálint Wenner, Gergő Rák, Konrad Mebert

**Affiliations:** 1 Kiskunság National Park Directorate, Kecskemét, Hungary; 2 Centre for Ecological Research, HUN-REN, Debrecen, Hungary; 3 Institute of Metagenomics, University of Debrecen, Debrecen, Hungary; 4 Evolutionary Ecology Group, Hungarian Department of Biology and Ecology, Babeş-Bolyai University, Cluj-Napoca, Romania; 5 “Milvus Group” Bird and Nature Protection Association, Tîrgu Mureș, Romania; 6 Department of Systematic Zoology and Ecology, Eötvös Loránd University, Budapest, Hungary; 7 Faculty of Science and Technology, Juhász-Nagy Pál Doctoral School, University of Debrecen, Debrecen, Hungary; 8 IDECC Institute of Development, Ecology, Conservation and Cooperation, Rome, Italy; 9 Birr, Switzerland

**Keywords:** Squamate, Reptiles, Activity budget, Climatic shift, Global warming

## Abstract

Climate change threatens species adapted to cool alpine environments, particularly ectotherms like reptiles. Small-sized grassland specialist vipers inhabit such environments in Eurasia and are highly susceptible to overheating and dehydration as global temperature rises. This study modelled activity restriction times, defined as hours when environmental temperatures exceed the thermal tolerance (i.e. not available for essential activities) of the species, for 20 grassland viper taxa to assess climate change impacts. Under future conditions, hours of activity restriction are projected to increase by 21% by the SSP1-2.6 scenario, and by 52.1% by the SSP5-8.5 scenario. Elevation and latitude significantly influenced restriction time changes, with high-altitude and northern populations predicted to be most affected. The taxa *Vipera graeca* and *Vipera ursinii moldavica* are expected to experience the greatest increase in restriction times. Despite warmer conditions potentially increasing hours within preferred thermal ranges, vipers are unlikely to exploit lower-elevation habitats due to competition and ecological constraints. These findings emphasise the urgent need for conservation strategies, including habitat preservation and connectivity, to mitigate the adverse effects of climate change on grassland vipers, particularly the most vulnerable populations.

## Introduction

Climate change is a pressing global issue, pushing many species to their thermal tolerance limits [1, 2]. Climate change threatens ectothermic animals that depend on environmental heat sources to regulate their body temperature by balancing physiological and behavioural mechanisms [3]. As environmental temperatures rise, many species encounter conditions that exceed their physiological thermal limits [4, 5]. Ectotherms may respond in either of three different ways: developing adaptations, shifting geographic ranges, or going locally extinct [6, 7]. These responses depend on the speed of environmental changes relative to the capacity for dispersal and physiological or behavioural adjustments. Understanding whether these shifts and adaptations can occur rapidly enough is an urging question in current biology, with implications for biodiversity conservation [8, 9].

Reptiles, as ectotherms, are highly sensitive to changes in their thermal environment. Elevated temperatures disrupt the balance between thermal preferences and environmental conditions, impacting critical life history processes such as foraging and reproduction [10]. Exceeding thermal maxima is predicted to reduce activity times and fitness, ultimately influencing reproductive success and population density [11–13]. In temperate climate zone, where our study species occur, warm winters can lead to intermittent activity during hibernation, such as attempts at foraging. However, these activities may result in energy deficits due to higher metabolism rate during hibernation and increased predation risk [14]. Additionally, while warmer climates may extend the active season, they simultaneously increase the frequency of extreme temperatures above critical thermal limits [15]. Such changes often force reptiles into suboptimal microhabitats, increasing predation risks and worsening fitness consequences. These individual-level effects can scale up to alter population dynamics and geographic distributions. In addition, extreme climatic events, including heatwaves can have severe long-term effects on populations [16].

Despite increasing awareness of the impacts of climate change on ectotherms, significant knowledge gaps remain regarding population and individual-level consequences. Changes in activity patterns due to exceeding thermal limits could contribute to declines in population size [11, 13, 17]. However, most studies rely on correlative approaches, using occurrence data to predict gains and losses in species distributions, but these do not incorporate the physiological and behavioural mechanisms that drive species responses, limiting our ability to accurately forecast the impacts of climate change on activity budgets, i.e. the duration of thermally favourable conditions available for activity [4, 18]. These areas are open for further exploration and are essential for identifying threats and informing conservation strategies aimed at mitigating the effects of climate change on vulnerable species.

Reptiles are exposed to increasing threats worldwide, with about 20% of species at risk of extinction due to the synergistic interaction of factors including habitat loss, fragmentation and degradation, environmental pollution, and the spread of diseases, among others [19]. Evidence-based conservation strategies for reptiles in the face of climate change necessitate the identification of threatened populations and priority areas [20]. Following these priorities, there is an increasing demand for nature-based solutions (NbS), which leverage the protection, restoration, and sustainable management of ecosystems to address societal challenges, that have emerged as a priority for effectively mitigating the adverse impacts of climate change while enhancing benefits for people [21, 22]. However, conservation actions could also be implemented to increase habitat size and quality to support higher population sizes [23], which would be crucial for maintaining genetic diversity and providing the genetic basis for potential adaptations to a changing environment. Additionally, enhancing habitat connectivity could facilitate species dispersal, enabling reptiles to track shifting suitable habitats as climate conditions evolve. Accurate knowledge of microclimatic conditions is essential for predicting the impacts of climate change and developing conservation strategies. The findings can be utilized for species conservation measures, such as improving the microclimatic conditions of habitats [24]. Addressing existing knowledge gaps through targeted research can further refine these strategies, ensuring they are grounded in a comprehensive understanding of species-specific responses to climate change.

This study aims to predict changes in the activity budget of a functional group of true vipers (*Vipera* spp.) living in open grassland ecosystems using mechanistic modelling of operative temperatures under current and future climate scenarios. By integrating thermal tolerance data with climatic projections, we identify populations at the highest risk of activity restriction. The results will provide actionable insights for conservation strategies, enhancing our understanding of the impacts of climate change on the thermal ecology of these threatened species.

## Materials and methods

### Study species

**Fig. 1:**
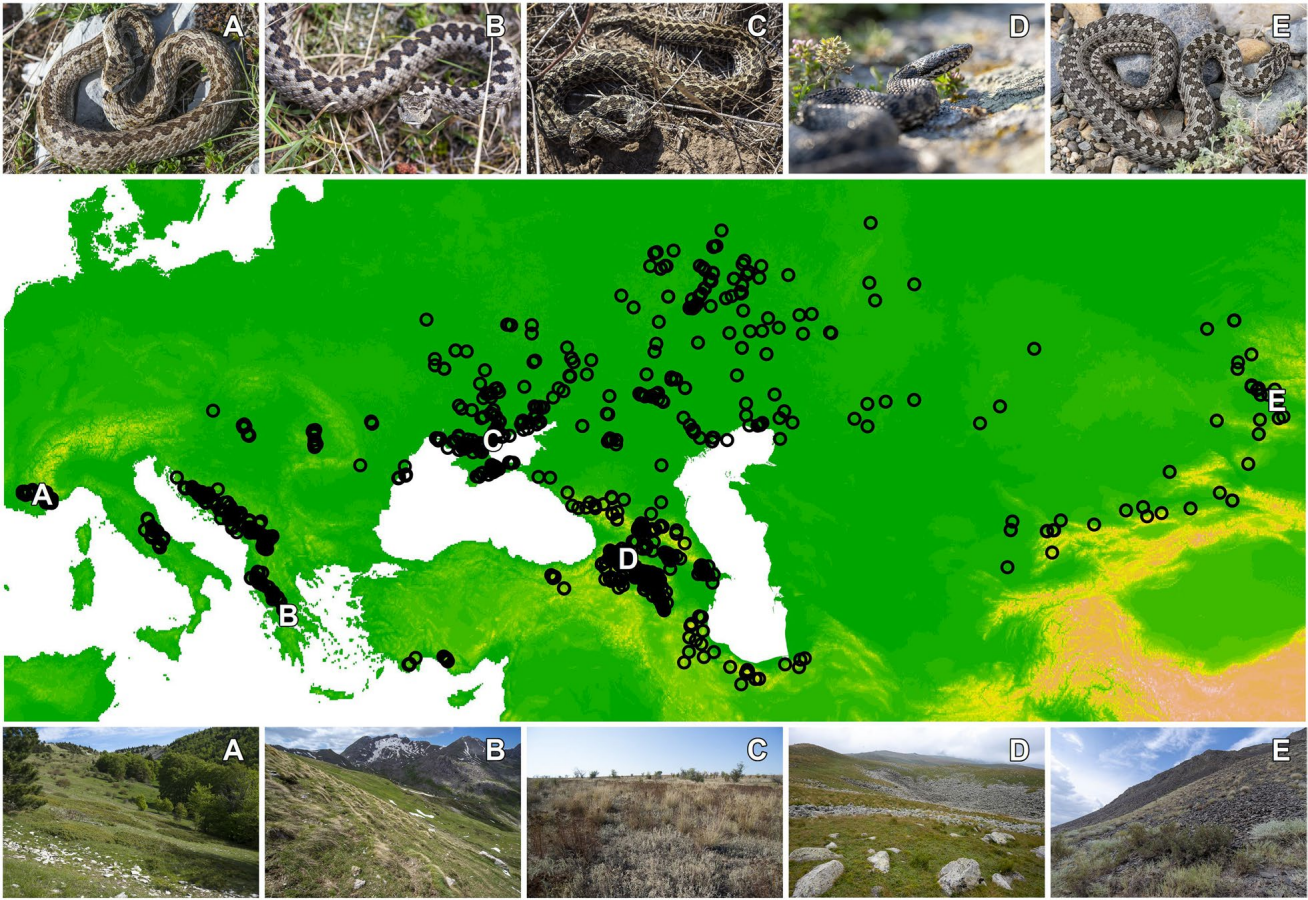
Occurrence records of grassland vipers used in this study and examples of grassland vipers and their habitats (**A** *Vipera ursinii ursinii*, France; **B** *Vipera graeca*, Greece, **C** *Vipera renardi renardi*, Ukraine; **D** *Vipera darevskii*, Georgia; **E** *Vipera renardi altaica*, Kazakhstan; pictures by E. Mizsei)

Grassland vipers represent a functional group of true vipers belonging to the monophyletic subgenus *Acridophaga* within the genus *Vipera*. In this article, following some recent works [25–28], we refer to grassland vipers as a guild encompassing numerous phylogenetically distinct taxa of the meadow vipers (*Vipera ursinii* complex) and steppe vipers (*V. renardi* complex) and the species *V. dinniki*, *V. graeca*, *V. darevskii* and *V. anatolica*. These are recognised as evolutionarily significant units (ESUs) due to the distinct levels of divergence and evident allopatric speciation patterns [29–34]. Grassland vipers occupy a wide range of the Palaearctic grassland biome including semi-deserts in Central Asia, lowland steppes at higher latitudes and alpine grasslands above the tree line in the Mediterranean and Central Asian mountain chains (Fig. 1). However, this functional group of snakes is not fully monophyletic, because of the taxa *V. kaznakovi*, which mostly inhabits closed, temperate rainforest and deciduous forest habitats from lowlands to mid-elevations and rarely grasslands, which is most likely a unique adaptation within this complex, thus we have excluded this taxa from the study, due to its unknown thermal behaviour and distinct habitat preferences [35].

Grassland vipers often occur in small, isolated populations, rendering them highly sensitive to habitat fragmentation and degradation [36]. While some ESUs are not currently considered threatened, the majority faces varying levels of threats, ranging from vulnerable to critically endangered by risk of extinction [20, 37, 38]. Cold-climate-adapted Mediterranean species such as *V. ursinii* and *V. graeca* are among the most threatened snakes in Europe due to their narrow ecological niches and vulnerability to warming climates [20, 39, 40].

### Data

We utilized voluntary thermal maximum (*VT*_*max*_) values measured or predicted for grassland vipers [27] to parameterize an ectotherm model. Occurrence records (n = 4,032) were compiled from published datasets and our research group’s field observations, covering the geographic ranges of grassland vipers. Monthly air temperature data at a 30″ spatial resolution (~ 720 × 930 m grid) were extracted from the WorldClim 2.1 database for both current (1970–2000) and future (2081–2100) conditions. To characterize future climate scenarios, we selected three Global Climate Models (GCMs, HadGEM3-GC31-LL, IPSL-CM6A-LR, and MIROC6) under two Shared Socioeconomic Pathways (SSPs): SSP1-2.6 (low-emission scenario) and SSP5-8.5 (high-emission scenario). These scenarios capture a range of possible outcomes, from ambitious mitigation efforts to continued high emissions.

### Modelling

We estimated operative or environmental temperatures (*T*_*e*_) and calculated activity restriction times using mechanistic models in the NicheMapR package [41]. These process-based models solve coupled energy and mass balance equations to establish an explicit link between the organism’s requirements and the availability of environmental resources. Operative temperature is the environmental temperature that is available to an individual at different times during thermoregulation [42]. We calculated the restriction time of activity as the number of hours during a full year (24 × 365) when *T*_*e*_ exceeded *VT*_*max*_ resulting in the hours of restriction, *h*_*r*_ [43]. Previous studies have demonstrated that NicheMapR produces highly accurate and reliable estimates of operative temperatures across various environments and taxa, with generally low bias and strong correlation to field measurements (e.g. [44–46]).

To get *T*_*e*_ covering all occurrence locations (n = 1420 grid cell at the spatial resolution of 30″) of grassland vipers, we used a microclimate model to establish a simulated environment and an ectotherm model to predict *T*_*e*_. As the default temperature data in NicheMapR has a low spatial resolution (10 × 10 km grid), we used the current (1970–2000) monthly mean air temperature at 30″ resolution and extracted the local values for each viper occurrence grid cell and set the warm parameter to the difference of the fine resolution and the default monthly temperature values to establish a corrected microclimate model for current climatic conditions. In this simulated microclimate environment, we fitted an ectotherm model for each location and supplied the local viper traits such as weight, shape of the animal (cylinder), and diurnality. We fitted the ectotherm model as “dead” (parameter live = 0), because we were interested in getting *T*_*e*_ measurements, meaning the body temperature of a non-thermoregulating individual (no heat-seeking or avoidance by moving). In the next step, we extracted the *T*_*e*_ estimates from the ectotherm model output and counted the number of hours when *T*_*e*_ exceeded the *VT*_*max*_ (= *h*_*r*_). To estimate *h*_*r*_ for future climate scenarios, we used the warm parameter similarly as above using the monthly air temperatures of the future data sets (n = 6 = 3 GCM × 2 SSP). We repeated this procedure for each location to map *h*_*r*_ for all occurrence locations of grassland vipers.

After getting *h*_*r*_ for each location, we calculated the median of *h*_*r*_ (50th percentile) and interquartile range (5th and 95th percentiles) as confidence interval (CI) of estimates for each taxon and climate scenario and the difference between future and current climate restriction times to predict the change in activity budget of grassland vipers. We evaluated the estimates by comparing the values of *h*_*r*_ of current and future climates by Mann–Whitney test. To assess the influence of latitude and elevation we fitted linear models. All data operations and statistical analyses were conducted in the R 4.4.2 statistical environment [47].

## Results

At current climatic conditions, the cross-taxa median restriction time (*h*_*r*_) of all grassland vipers was *h*_*r*_ = 1168 (CI 558–1531) hours. For future conditions, regarding the SSP1-2.6 scenario, it is expected to increase to *h*_*r*_ = 1413 (CI 836–1760) hours, or based on the SSP5-8.5 scenario, to *h*_*r*_ = 1776 (CI 1224–2150) hours. That means *h*_*r*_ will increase by 21% in the SSP1-2.6 and 52.1% in the SSP5-8.5 scenario.

**Fig. 2:**
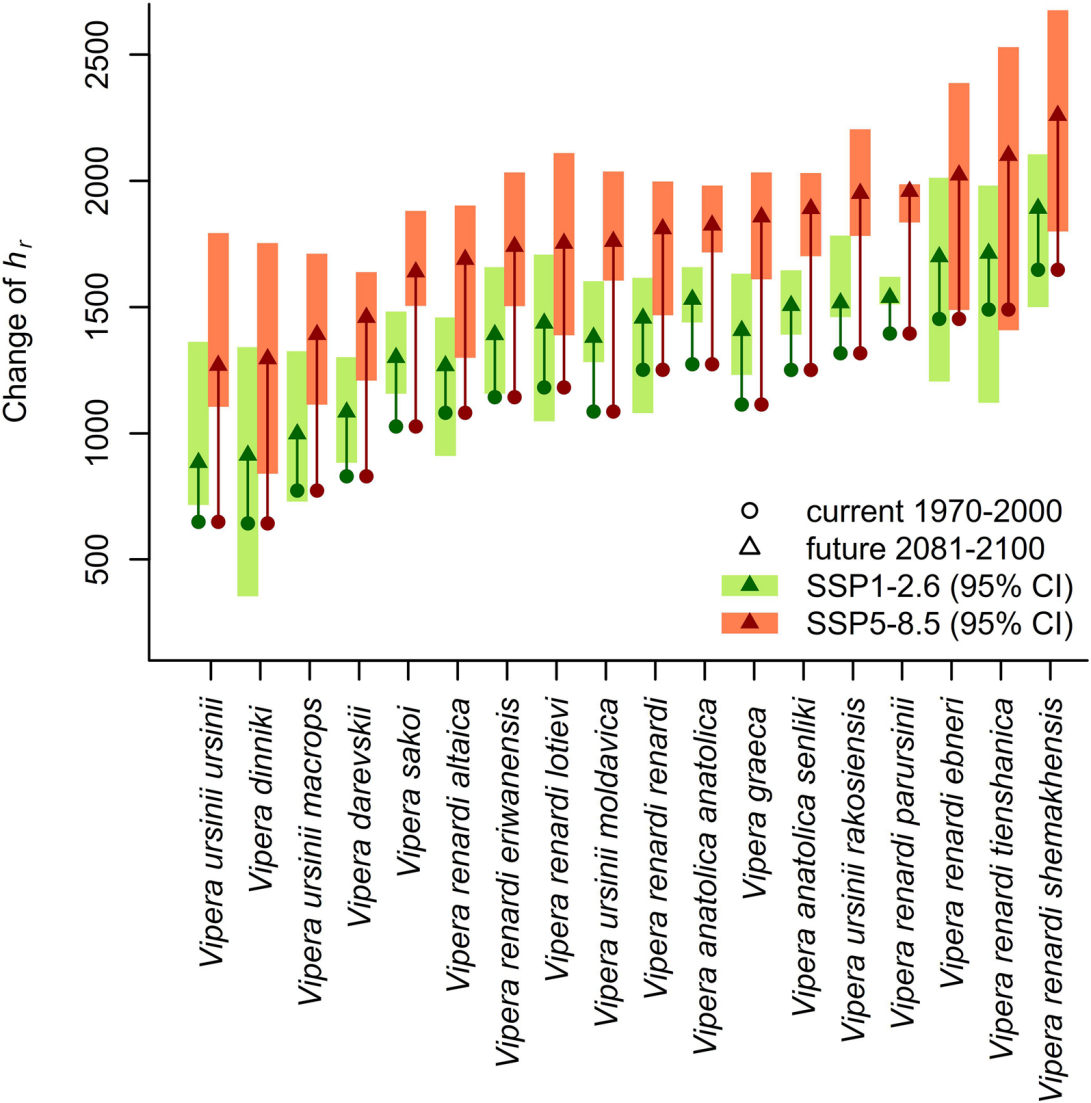
Averaged hours of restriction (*h*_*r*_) of grassland vipers according to two climate change scenarios. CI ranges are indicated only for future *h*_*r*_ estimates to enhance visibility

We compared the current and future values of *h*_*r*_ in each taxon because some overlap of values of the highest current *h*_*r*_ and lowest future *h*_*r*_ can be observed (Fig. 2). We found that in the case SSP1-2.6 scenario future *h*_*r*_ will be significantly higher than the current *h*_*r*_ (*p* < 0.001) in all taxa, except for *Vipera renardi lotievi* (current *h*_*r*_ = 1185 [CI 830–1456], SSP1-2.6 future *h*_*r*_ = 1436 [CI 1047–1708]) and *V. r. parursinii* (current *h*_*r*_ = 1395 [CI 1374–1397], SSP1-2.6 future *h*_*r*_ = 1538 [CI 1511–1620]). In the SSP5-8.5 scenario, the future *h*_*r*_ will be significantly higher than the current *h*_*r*_ in all taxa (*p* < 0.0001). The highest increase of *h*_*r*_ is expected for the taxa *V. graeca* and *V. ursinii moldavica* (Fig. 2).

**Fig. 3:**
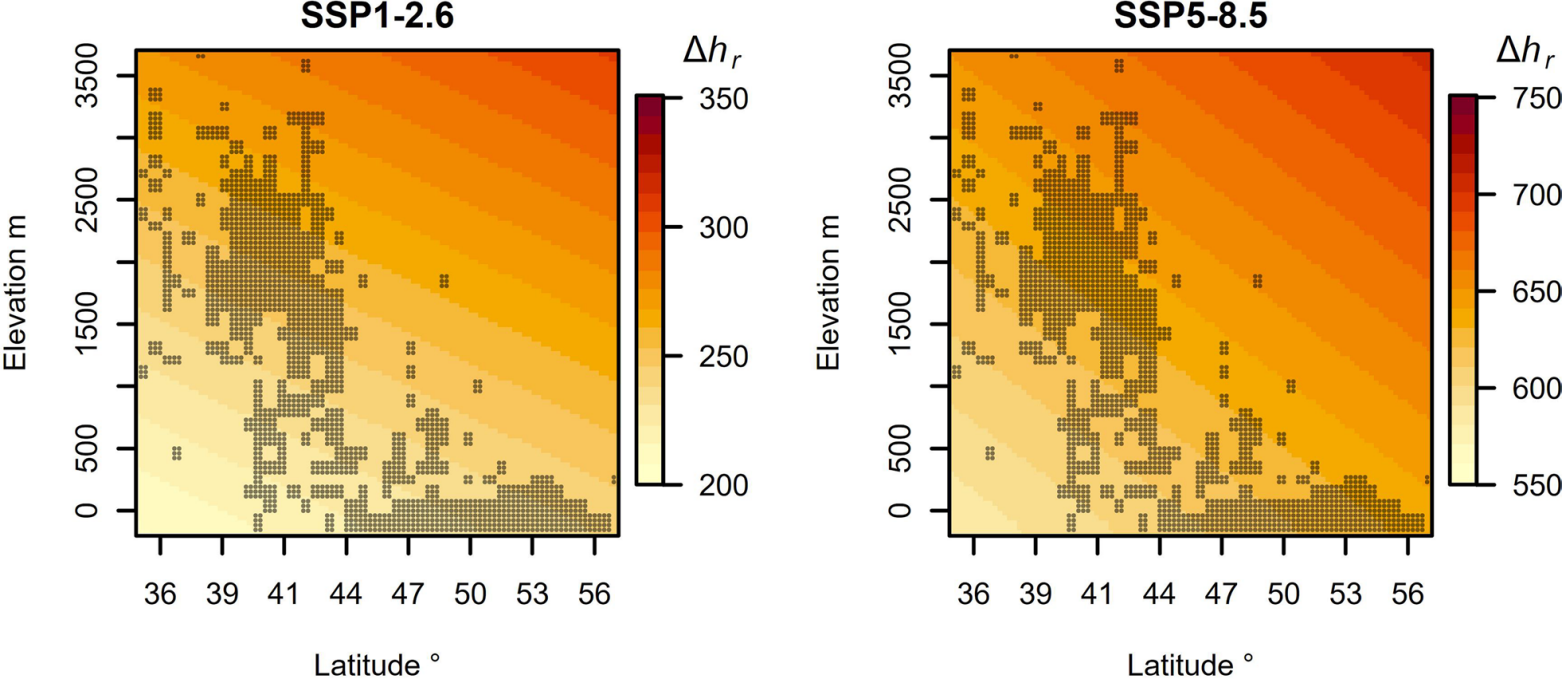
Generalized prediction of change in hours of restriction from current to future timescales (Δ*h*_*r*_). Small dots indicate the current occurrence of grassland vipers at a given latitude and elevation

**Fig. 4:**
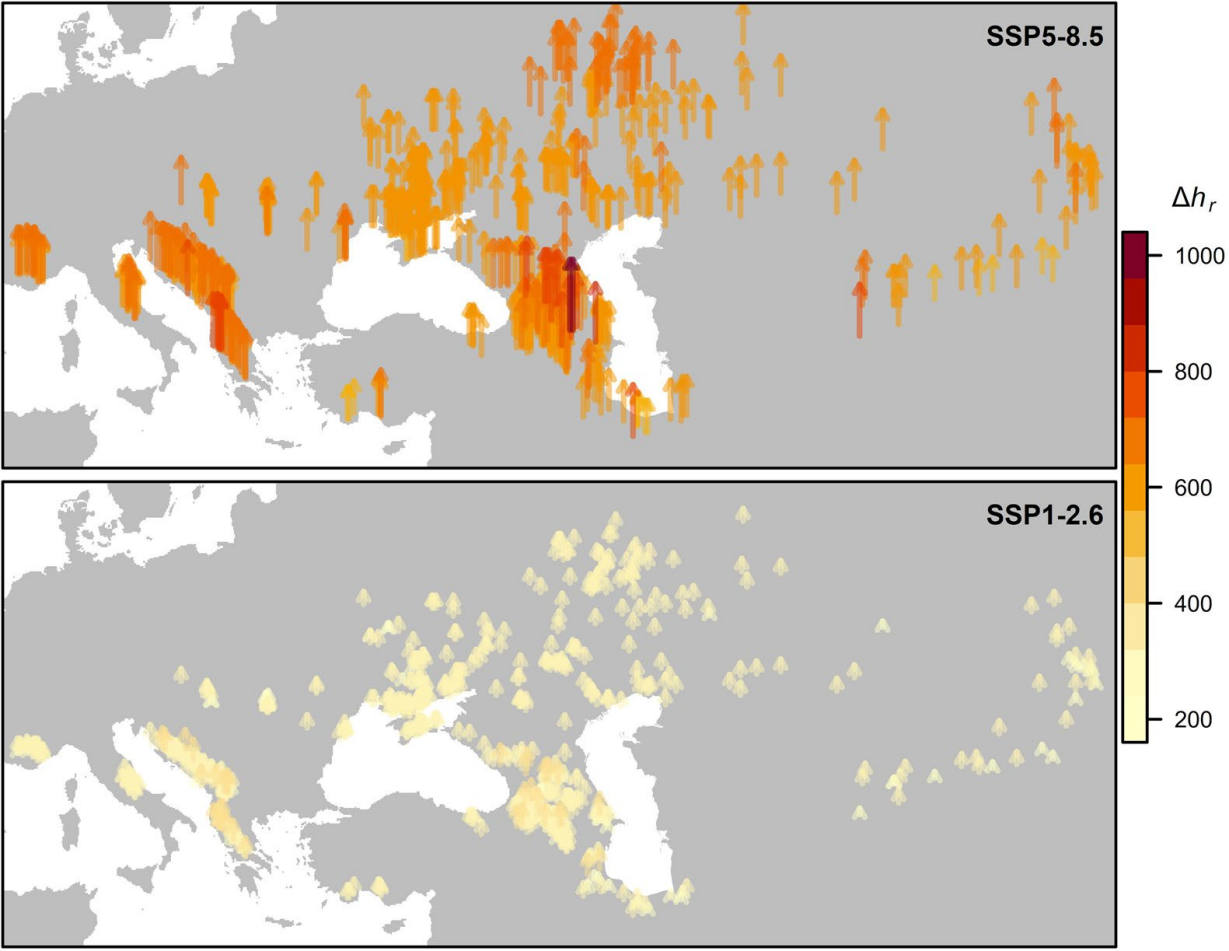
The spatial pattern of change in hours of restriction from current to future timescales (Δ*h*_*r*_). The length and colour of arrows are proportional to Δ*h*_*r*_

We explored the spatial patterns of *h*_*r*_ change from current to future timescales (Δ*h*_*r*_). Elevation and latitude both significantly positively influenced Δ*h*_*r*_ (Fig. 3): for the SSP1-2.6 scenario, latitude (β = 1.59 ± 0.35 [SE], t = 4.59, *p* < 0.0001) and elevation (β = 0.02 ± 0.001 [SE], t = 12.54, *p* < 0.0001) significantly increased Δ*h*_*r*_; for the SSP5-8.5 scenario we observed a similar pattern (latitude: β = 2.55 ± 0.54 [SE], t = 4.66, *p* < 0.0001; elevation: β = 0.02 ± 0.002 [SE], t = 8.12, *p* < 0.0001). The Δ*h*_*r*_ is expected to be greater at higher elevations (i.e. alpine habitats) and latitudes (i.e. northern lowland grasslands), with populations at high elevations to be the most affected (Fig. 4).

## Discussion

Our study estimated significant increases in activity restriction times (*h*_*r*_) for grassland vipers under future climate scenarios, aligning with broader research on ectotherms facing thermal constraints due to climate change. These species, reliant on external heat sources for thermoregulation and proper physiological processes, are increasingly being pushed toward their thermal tolerance limits as temperatures rise [1, 3]. The narrow thermal preferences and small size of grassland vipers exacerbate their vulnerability, particularly in open habitats with limited thermal refugia [20, 48]. The pronounced increase in *h*_*r*_ observed at high-altitude and northern populations parallels patterns in ectotherms living in montane areas or high latitudes, further emphasizing the critical need for targeted interventions [6, 49]. These findings contribute to the growing body of evidence that climate-induced thermal stress poses severe challenges to the ectothermic biodiversity and underscores the importance of region-specific conservation efforts.

This study adopted a mechanistic modelling approach to assess how changes in thermal landscapes impact activity budgets, diverging from the correlative models frequently used in similar research [4, 50, 51]. By incorporating physiological data such as *VT*_*max*_ and body weight, we provided precise and biologically grounded predictions of activity restriction. This methodology complements previous work on dendrobatid frogs, where physiological thermal niches were critical for predicting biological stress for species from different elevation zones during global warming [7]. Extending this approach to vipers enhances our understanding of species-specific responses and offers a robust framework for evaluating the impacts of thermal constraints across diverse taxa.

The projected increases in *h*_*r*_ present multifaceted challenges to grassland vipers, as restricted activity reduces opportunities for foraging, mating, and thermoregulatory activities, such as digesting and shedding. These limitations, worsened by habitat fragmentation and degradation, pose direct threats to population viability. Our findings stress the importance of preserving large, contiguous habitats to sustain genetic diversity and adaptive potential [20, 52]. Furthermore, enhancing habitat connectivity can facilitate range shifts and mitigate the demographic impacts of fragmented distributions. Conservation actions should prioritize high-altitude and northern regions, where the combined effects of thermal stress and restricted activity are most pronounced. This study also highlights the need for transnational cooperation to develop comprehensive strategies addressing these spatially and temporally variable threats.

While this study provides critical insights, some limitations warrant consideration. The geographic distributions of some grassland viper taxa remain inadequately mapped, preventing precise predictions for under-studied populations [25, 26, 53]. Additionally, *VT*_*max*_ and body weight were modelled for certain taxa rather than directly measured, introducing potential uncertainties in the physiological parameters of our models. Another limitation stems from the inherent unpredictability of future climate trajectories, with different emission scenarios yielding varying outcomes. Moreover, the long-term impacts of restricted activity on fitness and reproductive success remain poorly understood. Addressing these gaps through field research, refined physiological measurements, and long-term monitoring will enhance the reliability of predictions and support the formulation of effective conservation strategies.

An important question arising from our findings is why grassland vipers do not currently occupy the hotter, low-elevation habitats that are already available and likely offer a greater number of hours within their preferred thermal range (*T*_*set*_). These warmer grasslands may already present thermal conditions comparable to those predicted for the vipers’ existing habitats under future climate scenarios. This raises the possibility that, under warming conditions, current populations may face local extinction rather than adapt in situ, especially if physiological or behavioural changes allowing such habitat shifts were possible and have not occurred during past, slower-paced climate change. Competition likely plays a significant role, with grassland vipers being more successful in environments to which other reptiles are less adapted [20, 54]. This aligns with findings by Lucchini et al. [55], who demonstrated that climatic niche differentiation is a critical factor in the distribution of European vipers, shaped by both historical adaptations and ecological interactions. The thermal niches available at lower elevations likely overlap with a larger diversity of other verterbrate species, intensifying competitive, but also predatory pressure from a larger set of colubrids, birds, and mammals and thus, reducing habitat suitability for vipers. Understanding how future climate scenarios will alter these dynamics is essential for predicting shifts in viper distributions and informing conservation strategies.

Future research should explore the relationship between restricted activity budgets and fitness outcomes, particularly regarding impacts on reproduction and survival. Investigating how historical shifts in activity times influenced population demography could provide context for current and future challenges. Identifying populations with broader thermal niches or thermoregulatory adaptations will help prioritize conservation efforts for the populations or taxa that are most vulnerable. Additionally, integrating genomic studies with mechanistic models and long-term ecological monitoring will enhance our understanding of adaptive capacity and phenotypic plasticity in response to climate change. These efforts would inform targeted interventions to mitigate the impacts of warming climates on grassland vipers and other threatened ectotherms.

This study aimed to assess the impact of climate change on grassland viper activity budgets by predicting increases in activity restriction times across their range. Using mechanistic models informed by physiological and climatic data, we demonstrated that *h*_*r*_ is projected to increase significantly, particularly in high-altitude and northern populations. These findings underscore the profound implications for individual fitness, and population dynamics, highlighting the urgency of conservation interventions. By identifying vulnerable populations and regions, our study provides a foundation for prioritizing conservation actions. Integrating physiological and ecological insights into conservation planning will be essential to address the complex challenges posed by climate change.

## Data Availability

Codes for statistical analysis and the data included in the study are archived at Zenodo repository (10.5281/zenodo.15616045). Distribution coordinates were generalised to a lower spatial resolution to protect the precise locations of viper populations, as these are considered sensitive data.
